# RNA-seq comparative analysis of Peking ducks spleen gene expression 24 h post-infected with duck plague virulent or attenuated virus

**DOI:** 10.1186/s13567-017-0456-z

**Published:** 2017-09-13

**Authors:** Tian Liu, Anchun Cheng, Mingshu Wang, Renyong Jia, Qiao Yang, Ying Wu, Kunfeng Sun, Dekang Zhu, Shun Chen, Mafeng Liu, XinXin Zhao, Xiaoyue Chen

**Affiliations:** 1 Avian Disease Research Center, College of Veterinary Medicine, Sichuan Agricultural University, Wenjiang, Chengdu, 611130 People’s Republic of China; 2 Institute of Preventive Veterinary Medicine, Sichuan Agricultural University, Wenjiang, Chengdu, 611130 People’s Republic of China; 3 Key Laboratory of Animal Disease and Human Health of Sichuan Province, Wenjiang, Chengdu, 611130 People’s Republic of China

## Abstract

Duck plague virus (DPV), a member of alphaherpesvirus sub-family, can cause significant economic losses on duck farms in China. DPV Chinese virulent strain (CHv) is highly pathogenic and could induce massive ducks death. Attenuated DPV vaccines (CHa) have been put into service against duck plague with billions of doses in China each year. Researches on DPV have been development for many years, however, a comprehensive understanding of molecular mechanisms underlying pathogenicity of CHv strain and protection of CHa strain to ducks is still blank. In present study, we performed RNA-seq technology to analyze transcriptome profiling of duck spleens for the first time to identify differentially expressed genes (DEGs) associated with the infection of CHv and CHa at 24 h. Comparison of gene expression with mock ducks revealed 748 DEGs and 484 DEGs after CHv and CHa infection, respectively. Gene pathway analysis of DEGs highlighted valuable biological processes involved in host immune response, cell apoptosis and viral invasion. Genes expressed in those pathways were different in CHv infected duck spleens and CHa vaccinated duck spleens. The results may provide valuable information for us to explore the reasons of pathogenicity caused by CHv strain and protection activated by CHa strain.

## Introduction

After firstly reported in Netherlands at 1923, duck plague (DP) was rapidly spread around the world [1]. In China, the DP was not suspected until 1958 and the outbreak of DP results in a devastated hit to duck industries in China, one of the largest duck breeding countries in the world, due to DP’s high mortality [2, 3]. The disease is caused by duck plague virus (DPV), a member of alphaherpesvirus subfamily, which is a double-stranded DNA virus composed with capsid, tegument and envelope [4]. DPV Chinese virulent strain (CHv), which was isolated from dead infected ducks in China, is highly pathogenic and could induce massive petechial hemorrhages in parenchymal organ, lymphoid and digestive tract and causes massive ducks death [5–8]. To control this disease on duck farms, attenuated DPV vaccines, such as CHa, have been put into service against duck plague with billions of doses in China each year [9].

Compared to other Herpesviruses, the development of DPV researches, particularly in viral gene functions and virus-host interplay, undergoes a slow process. After the first reports of DPV highly virulent strain and attenuated strain genomes, the studies of gene structure and functions have been spring up. DPV highly virulent strain genes, such as capsid protein genes UL19, UL35 tegument protein genes UL16, UL51 and envelope glycoprotein genes like UL44, US7, US8 were well studied [10–16]. Furthermore, it has been reported that the five ORFs, including UL2, UL12, US10, UL47 and UL41, might represent DPV virulence factors [17]. However, although exploring basic gene mechanisms of highly virulent DPV strains and attenuated DPV strains on genomic level is indeed helpful, it still lacks systemic and comprehensive studies on nature host duck biological processes when infect with DP virus. Performed works reported that DPV virulent strain conducted a latent infection following a primary infection in trigeminal ganglion and lymphoid tissues including PBL and spleen [18], whereas how the virus processes a latent infection and what the mechanisms of latency infection have not been clear. Meanwhile, although Yuan observed programmed cell death on lymphocytes after infection of highly virulent DPV strain, and the result indicated membrane proteins of DPV could alter characteristics of apoptotic cells in duck embryo fibroblast culture [19]. However, no further studies about DPV-induced apoptosis were observed. Moreover, as we all know, the processes for virus to entry into a cell is critical to initiate their whole life cycles, however, we have little information about DPV entry [20–22]. Although genes involved in other herpesvirus entry such as glycoprotein gB and gD were well studied, it is still a puzzle part that whether these genes are necessary in DPV entry or not [23, 24].

In this study, with the help of RNA-seq platform we explored a bioinformatics analysis on duck spleen gene expression for the first time after highly virulent DPV CHv strain and attenuated DPV CHa strain infection at post-infected 24 h. Previous study indicated that attenuated DPVs were rapid distribution and proliferation in duck’s tissues 12 h post-inoculation, however, copies of attenuated DPV were dramatic decline in duck’s tissues 24 h post-infection (hpi), which means that attenuated DPV could rapidly invoke host immune response and inhibit DPV proliferation in the early stage of inoculation [6]. Thus, 24 h was chosen as a samples collection time point to explore reasons of protection stimulated by attenuated DPV in short time after infection. In order to detect accurate biological differences from sequencing data, the setting of multiple biological replicates and the meticulous analysis of statistic were performed. Furthermore, high quality reads mapping was presented by using a paired-end mRNA sequencing approach. Eventually, 748 differentially expressed genes (DEGs) in CHv infected group and 484 DEGs in CHa vaccinated group were selected. Gene pathway analysis of DEGs highlighted several valuable biological processes involved in host immune response, cell apoptosis and viral invasion. Genes expressed in those pathways were different in CHv infected duck spleens and CHa vaccinated duck spleens. Overall, our findings generated in this work firstly provided a detailed view of global changes in duck spleen gene expression when infected with either highly virulent DPV CHv strain or attenuated DPV CHa strain at 24 hpi and the results may provide valuable information for us to explore the reasons of pathogenicity caused by CHv strain and protection activated by CHa strain.

## Materials and methods

### Virus and experiment design

Highly virulence DPV CHv strain and attenuated modified vaccine DPV CHa strain used in this study were obtained from the Key Laboratory of Animal Disease and Human Health of Sichuan Province. Twenty-seven Peking ducklings were conducted from a DPV-free farm where vaccination against DPV was not implementation. Three experimental groups, including CHv infected group, CHa vaccinated group and normal saline intramuscular injected control group, were explored in this study. Each experimental group contained three biology repeats and each repeat included three ducks. CHv-infected and CHa-vaccinated groups were inoculated using intramuscular administration (4.1 × 10^8^ copies CHv and CHa, respectively). Ducks were euthanized to collect spleen samples at 24 hpi. The samples were rapidly kept in liquid nitrogen for further experiments.

### Library preparation for RNA-seq

Total RNA was isolated from spleen samples by TRIzol extraction (Invitrogen, CA, USA) following the manufacturer’s instructions. RNA purity was checked by using the NanoPhotometer^®^ spectrophotometer (IMPLEN, CA, USA) and its concentration was measured using Qubit^®^ RNA Assay Kit in Qubit^®^ 2.0 Flurometer (Life Technologies, CA, USA). RNA integrity was assessed using the RNA Nano 6000 Assay Kit of the Bioanalyzer 2100 system (Agilent Technologies, CA, USA). A total amount of 3 μg RNA per sample was used as input material for the RNA sample preparations. Sequencing libraries were generated using NEBNext^®^ UltraTM RNA Library Prep Kit for Illumina^®^ (NEB, USA) following manufacturer’s recommendations and index codes were added to attribute sequences to each sample. Briefly, mRNA was purified from total RNA using poly-T oligo-attached magnetic beads. Fragmentation was carried out using divalent cations under elevated temperature in NEBNext First Strand Synthesis Reaction Buffer (5×). First strand cDNA was synthesized using random hexamer primer and M-MuLV Reverse Transcriptase (RNase H^-^). Second strand cDNA synthesis was subsequently performed using DNA Polymerase I and RNaseH were converted into blunt ends via exonuclease/polymerase activities. After adenylation of 3′ ends of DNA fragments, NEBNext Adaptors with hairpin loop structure were ligated to prepare for hybridization. In order to select cDNA fragments of preferentially 150–200 bp in length, the library fragments were purified with AMPure XP system (Beckman Coulter, Beverly, USA). Then 3 μL USER Enzyme (NEB, USA) was used with size-selected, adaptor-ligated cDNA at 37 °C for 15 min followed by 5 min at 95 °C before PCR. Then PCR was performed with phusion high-fidelity DNA polymerase, universal PCR primers and index (X) primer. At last, PCR products were purified (AMPure XP system) and library quality was assessed on the Agilent Bioanalyzer 2100 system. The clustering of the index-coded samples was performed on a cBot Cluster Generation System using TruSeq PE Cluster Kit v3-cBot-HS (Illumia) according to the manufacturer’s instructions. After cluster generation, the library preparations were sequenced on Hiseq-PE150 platform and 125 bp/150 bp paired-end reads were generated.

### Bioinformatics analysis of RNA-seq data

Clean reads were obtained to remove reads containing adapters, poly-N and low quality sequences in raw reads. Clean reads were calculated through Q20, Q30 and GC content to make sure data with high quality. All the downstream analyses were based on clean data with high quality. Paired-end clean reads were mapped to duck reference genome using TopHat v2.0.12. Reads numbers of each gene were counted through the HTSeq v0.6.1. In order to normalize the expression of each gene from different samples, FPKM (expected number of Fragments per kilobase of transcript sequence per Millions base pairs sequenced) was introduced. Genes with an expression level more than 1 FPKM were used for downstream analysis. To test the differential expression genes (DEGs) of two groups, CHv infected ducks and CHa vaccinated ducks (three biological replicates per group), the DESeq R package (1.18.0) was performed and genes with a *p-*value < 0.05 found by DESeq were assigned as differentially expressed.

### Gene ontology enrichment analysis

GOseq R package was used to perform gene ontology (GO) enrichment analysis of differential expression genes. GO terms with *p*-value < 0.05 were selected as significant enrichment.

### KEGG pathway analysis

KEGG is a database resource for understanding functions and utilities of the biological system, such as the cell, the organism and the ecosystem, from molecular-level information. We used KOBAS software to test the statistical enrichment of differential expression genes in KEGG pathways. KEGG pathways with *p*-value < 0.05 were selected as significant enrichment.

## Results

### Collection of duck spleen samples and construction of cDNA libraries after CHv and CHa infection

Transcriptome sequencing by RNA-seq was used to detect global changes of Peking duck spleen gene expression after CHv and CHa infection. Three experimental groups, including CHv infected group, CHa vaccinated group and normal saline intramuscular injected control group, were explored in this study. Each experimental group contained three biology repeats and each repeat included three ducks. Nine Peking duck spleen samples were collected and stored at liquid nitrogen until total RNA extraction and quality testing was done. Finally, nine cDNA libraries were performed.

### Differentially expressed genes after CHv and CHa infection

By using Hiseq-PE150 platform, a total of 571 million (571 134 310) raw data were produced across nine samples. To guarantee ideal results for following genomic mapping and differential gene change analysis, raw reads were filtered to remove low quality data with a total of 545 million (545 498 360) clean reads acquired, a mean of 61.23% of which mapped to duck reference genome **(**Table 1 **)**.

**Table 1:** **Number of reads of all bases detected using RNA-seq in DPV-infected and control ducks**

Library	Number of raw reads	Number of clean reads	Number of uniquely mapped reads	Percentage of reads mapped (%)
CHv-1	61 196 398	58 437 658	35 429 191	60.63
CHv-2	61 868 452	59 080 194	35 273 016	59.7
CHv-3	69 529 094	66 485 428	40 823 382	61.4
CHa-1	63 045 260	60 281 664	36 842 779	61.12
CHa-2	61 100 482	58 372 142	36 312 302	62.21
CHa-3	68 613 194	65 544 842	40 272 899	61.44
Control-1	64 108 500	61 235 756	37 298 283	60.91
Control-2	60 808 456	58 101 298	35 789 540	61.6
Control-3	60 864 474	57 959 378	35 637 789	61.49
Total	571 134 310	545 498 360	333 679 181	

**Figure 1:**
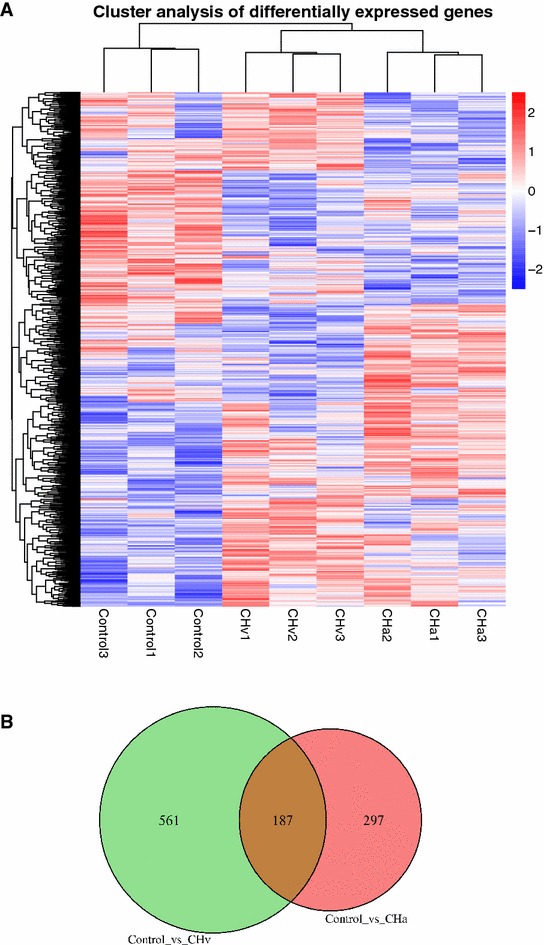
**Differentially expressed genes in different experimental conditions.** Heatmap is used to classify gene expression patterns under different experimental conditions and Venn diagram displays a global view on the numbers of differentially expressed genes. **A** Genes with similar expressed patterns were clustered and showed above in the heatmap. Intensity of color indicates gene expression levels that were normalized according to log_10_ (FPKM + 1) values. Red color represent high expression level genes and blue color represent low expression level genes. The three major clusters represent CHv infected duck group, CHa vaccinated ducks group and control group.** B** The overlap of differentially expressed genes of CHv vs Control and CHa vs Control. The number in the diagram indicated gene number refers to each comparison.

Heat map visually compares the whole level of different expression genes (DEGs) and classifies gene expression patterns in different treatment experimental groups under different experimental conditions. As showed in Figure 1A, notwithstanding genes harbored the same or similar expression pattern in CHv, CHa and control treatment groups, the regulation of gene expression was in an entirely different level. For exploring a global view on the change of duck gene expression between different experimental treatment groups, two paired comparisons (CHv vs control, CHa vs control) were performed. In all, RNA-seq analysis detected 748 and 484 genes, which were expressed at significantly different levels in CHv and CHa groups compared to control at a *p* value < 0.05, respectively **(**Figure 1B**)**. The purpose of this study is to explore differences of duck spleen gene expression at 24 h between high-virulence DPV CHv strain infected group and attenuated DPV CHa strain vaccinated group through high throughput RNA-seq analysis. In order to learn clearly and precisely about differentially expressed genes, venn analysis was utilized (Figure 1B) and there were three main parts of it. From the green part, there were 561 genes represented the numbers of genes only expressed in CHv infected ducks when compared to control and as for the red part, there were 297 DEGs that only expressed in CHa vaccinated ducks when compared to control. The intersection part with 187 DE genes were co-expressed genes in both CHv infected and CHa vaccinated groups when compared to control.

### Annotation of duck spleen DEGs based on GO analysis after CHv infection or CHa vaccination

To ensure differentially expressed genes function, gene ontology (GO) analysis was performed to categorize and annotate DEGs of all of three parts in Venn diagram into three groups including biological process (BP), cellular component (CC) and molecular function (MF), respectively. As shown in Table 2, a total of 2654 GO terms were assigned to DE genes only expressed in CHv infected group including 1806 BP terms, 511 MF terms and 341 CC terms. As for DE genes only expressed in CHa vaccinated group, a total of 1479 GO terms were annotated including 976 BP terms, 277 MF terms and 226 CC terms. Furthermore, DE genes expressed in both CHv infected group and CHa vaccinated group were categorized into GO terms with a total numbers of 1098 including 697 BP terms, 215 MF terms and 186 CC terms.

**Table 2:** **A summary of GO term numbers after CHv and CHa infection**

	GO terms-BP	GO terms-MF	GO terms-CC	Total terms
DEGs only express in CHv	1806	511	341	2658
DEGs only express in CHa	976	277	226	1479
DEGs expressed in CHv and CHa	697	215	186	1098

**Figure 2:**
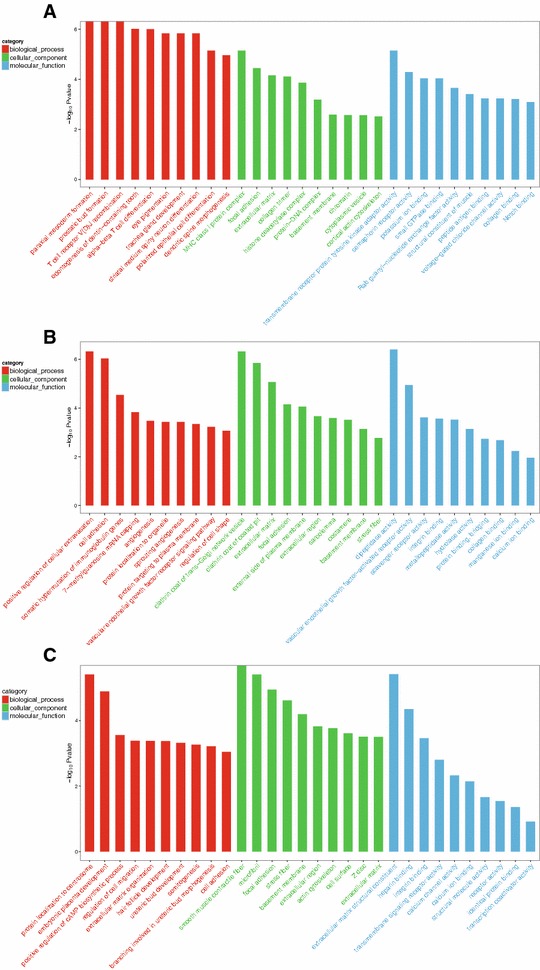
**Top 30 Gene ontology (GO) terms of DEGs expressed in CHv infected or CHa vaccinated group.** GO-terms were processed under three categories including cellular component (CC), molecular function (MF) and biological process (BP). Top 30 GO terms were selected according *p*-value < 0.05 **A** GO annotation of DEGs expressed only in CHv infected group **B** GO annotation of DEGs expressed only in CHa vaccinated group **C** GO annotation of DEGs expressed in both CHv infected group and CHa vaccinated group.

And then, in order to pick out the helpful and useful genes for further exploration, 30 significant GO terms were listed and each terms contained more than 2 genes (Figure 2). Top 30 GO terms were selected according *p*-value < 0.05. Top three significant GO terms of DEGs expressed in both CHv infected group and CHa vaccinated group were protein localization to centrosome (GO:0071539), smooth muscle contractile fiber (GO:0030485) and extracellular matrix structural constituent (GO:0005201). Top three significant GO terms of DEGs only expressed in CHv infected group were paraxial mesoderm formation (GO:0048341), MHC class I protein complex (GO:0042612) and transmembrane receptor protein tyrosine kinase adaptor activity (GO:0005068). Top three significant GO terms of DEGs expressed only in CHa vaccinated group were positive regulation of cellular extravasation (GO:0002693), clathrin coat of trans-Golgi network vesicle (GO:0030130) and dipeptidase activity (GO:0016805).

### Pathway analysis of DEGs based on KEGG after CHv and CHa infection

**Figure 3:**
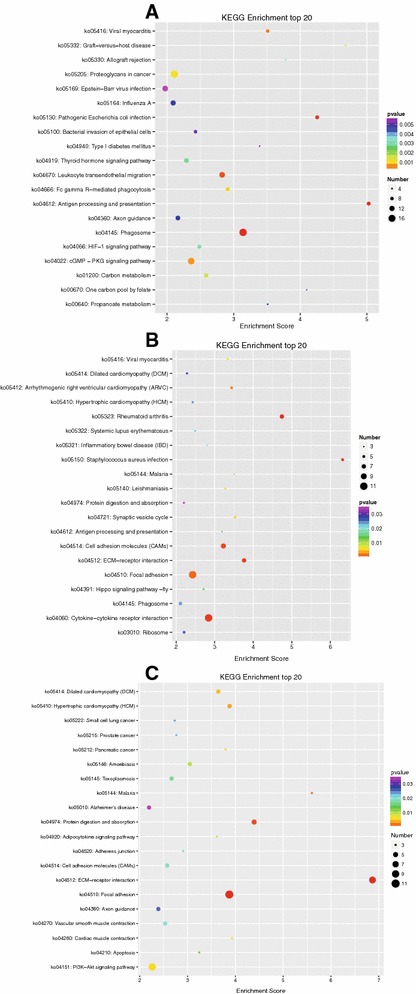
**Top 20 KEGG pathways in CHv infected or CHa vaccinated group.** **A** KEGG pathways of DEGs expressed in CHv infected group. **B** KEGG pathways of DEGs expressed in CHa vaccinated group. **C** KEGG pathways of DEGs expressed after CHv and CHa infection.

KEGG pathway analysis was used as a combination way to explore the function of DEGs. Top 20 enrichment KEGG pathways were listed in Figure 3 according to *p* value < 0.05. Four functional categories were identified to potentially play important roles associated with CHv infection including phagosome, antigen processing and presentation, leukocyte transendothelial migration and Fc gamma R-mediated phagocytosis. Three functional categories were identified to potentially play important roles associated with CHa vaccination including cytokine–cytokine receptor interaction, antigen processing and presentation as well as phagosome. Three functional categories were identified to potentially play important roles associated with both CHv infection and CHa vaccination including ECM-receptor interaction, focal adhesion as well as protein digestion and absorption. Putative functional roles and interactions of these genes involved in mediating host response of ducks are discussed in detail.

## Discussion

Duck plague (DP) is a disease that has been reported to cause significant economic losses on duck farms per year in China [3]. Although attenuated vaccine inoculation is an effective way for preventing this disease on duck industries, the potential risk of live attenuated vaccines of virulence return has raised more and more concern. Furthermore, the lack of effective therapeutic tools is another worry to control this disease spreading, indicating that it is urgent to not only give a deeply insight into pathogenesis mechanism of DPV highly pathogenic strain, but also give a profound understanding of protective mechanism of DPV attenuated vaccine strain. From our results, we performed RNA-seq technology for the first time and explored important information about host-viral interaction at post 24 h infection in which the ducks were infected by DPV highly pathogenic strain CHv and DPV attenuated vaccine strain CHa.

### Antigen processing and presentation

Cytotoxic T lymphocytes (CTLs) recognized virus-derived peptides presenting by MHC class I and MHC class II at cell surface is a critical immune monitor system and this high sensitive antigen processing and presentation system also becomes a prime target to escape host immune surveillance deployed by many viruses, particularly large DNA virus like herpesvirus [25]. Thus, antigen processing and presentation pathway annotated by KEGG analysis evoked our strong interests.

From results, we got differentially expressed genes related to antigen processing and presentation in KEGG pathway (Table 3). It was no surprise that at 24 hpi, duck spleen genes expression related to antigen processing and presentation had been changed when ducks infected no matter by CHv strain or by CHa strain. However, the results showed that there was huge diversity on the types of differentially expressed genes. CHv infection resulted in genes differential expression located to both MHC class I and MHC class II pathway, however, as for CHa infection, specific gene expression only changed in MHC class II pathway. Furthermore, from the results we could find that the MHC class II genes expressed were up-regulated in CHv infection group whereas down-regulated in CHa vaccinated group. It means that under the experiment condition in this study, the antigen processing and presentation process presented by highly virulence DPV strain CHv and attenuated DPV strain CHa is totally different in vivo.

**Table 3:** **Genes listed involved in multiple cell biological processes after CHv and CHa infection**

Gene ID	Gene name	Gene description	Source of DEGs
Antigen processing and presentation			
MHC-1 pathway			
101797502	MHC1	Major histocompatibility complex, class I	CHv vs control
101797680	TAP1	ATP-binding cassette, subfamily B (MDR/TAP), member 2	CHv vs control
101797091	TAP2	ATP-binding cassette, subfamily B (MDR/TAP), member 3	CHv vs control
101799108	β2m	Beta-2-microglobulin	CHv vs control and CHa vs control
MHC-II pathway			
101801019	LGMN	Legumain	CHv vs control
101803352	CD74	CD74 antigen	
101804125	MHC2	Major histocompatibility complex, class II	CHv vs control and CHa vs control
101803864	GILT	Gamma-inducible protein 30	
Cytokine-cytokine receptor interaction			
101799854	IFNGR1	Interferon gamma receptor 1	CHv vs control
101794299	CSF2RB	Cytokine receptor common subunit beta	CHv vs control
101791761	SF18	Tumor necrosis factor receptor superfamily member 18	CHv vs control
101799587	PDGFRB	Platelet-derived growth factor receptor beta	CHv vs control
101801232	SF21	Tumor necrosis factor receptor superfamily member 21	CHv vs control
101795038	CX3CL1	C-X3-C motif chemokine 1	CHa vs control
101790039	CCL19	C–C motif chemokine 19	CHa vs control
101802287	TGFB3	Transforming growth factor beta-3	CHa vs control
101793182	CNTFR	Granulocyte–macrophage colony-stimulating factor receptor alpha	CHa vs control
101797327	FLT1	FMS-like tyrosine kinase 1	CHa vs control
101792543	FLT4	FMS-like tyrosine kinase 4	CHa vs control
101795963	IL22RA2	Interleukin 22 receptor alpha 2	CHa vs control
101802521	IL15	Interleukin 15	CHv vs control and CHa vs control
101803777	TNFSF13B	Tumor necrosis factor ligand superfamily member 13B	CHv vs control and CHa vs control
101797830	EGFR	Epidermal growth factor receptor	CHv vs control and CHa vs control
Apoptosis			
101803461	PIK3CB	Phosphatidylinositol-4,5-bisphosphate 3-kinase catalytic subunit alpha/beta/delta	CHv vs control
101793887	IL3RB	Cytokine receptor common subunit beta	CHv vs control
101801064	IKKA	Inhibitor of nuclear factor kappa-B kinase subunit alpha	CHv vs control and CHa vs control
101794069	CASP6	Caspase 6	CHv vs control and CHa vs control
Viral invasion			
Cell surface-related receptor			
101796910	HSPG2	Heparan sulfate proteoglycan 2	CHv vs control
101791819	HS3ST5	Heparan sulfate glucosamine 3-*O*-sulfotransferase 5	CHv vs control
Cell cytoskeletal-related protein			
101805015	TUBA1C	Tubulin alpha-1C chain	CHv vs control
101805212	TUBA1B	Tubulin alpha-1B chain	CHv vs control
101801170	KIF14	Kinesin protein KIF14	CHa vs control
101793497	ACTA2	Actin, aortic smooth muscle	CHv vs control
101792376	ACTG2	Actin, gamma-enteric smooth muscle	CHv vs control and CHa vs control
101790019	AFAP1L2	Actin filament-associated protein 1-like 2	CHv vs control and CHa vs control
101799368	MYL9	Myosin light chain 9	CHv vs control and CHa vs control
101803563	MYH11	Myosin heavy chain 11	CHa vs control
Inner nuclear membrane-related protein			
101797969	LMNA	Lamin A/C	CHv vs control and CHa vs control

Virus antigens are degraded by proteasome in cytosolic origin in MHC class I presentation pathway and then are translocated into the endoplasmic reticulum (ER) by Transporter associated with Antigen Processing (TAP) to load onto newly synthesis MHC class I molecules [26]. In the ER, the MHC class I molecule is assembled from a heavy chain and a light chain called β_2_-microglobulin (β_2_m) [27]. The peptide inserts itself deep into the MHC class I peptide-binding groove and then ensure the stability of the complex [27]. The fully assembled molecule loaded with virus protein segments then release from the ER and travels via the Golgi apparatus to the plasma membrane for antigen processing and preparation to CD8^+^ T cells [28]. From our results, in CHv infected group, genes correlated to MHC class I molecule composition, such as MHC1 and β2m, and antigen processing transporters, such as TAP1 and TAP2, are up-regulated (Table 3). However, in CHa vaccinated group, only the expression of β2m gene was changed. Previous studies reported that herpesviruses could target each steps of MHC class I antigen presenting pathway to evade host immune response. A large number of proteins are encoded by herpesviruses to disturb MHC I antigen presentation and then prevent elimination of the target cell by CTLs. The mRNA of MHC class I high chain is degraded by host shut-off protein of herpes simplex virus (HSV) and bovine herpes virus (BHV-1) [29, 30]. Furthermore, immature MHC class I molecules are directed into cytosol from ER with the use of US10 protein of human cytomegalovirus (HCMV) and finally degraded by proteinase [31]. Moreover, with the help of ICP47, HSV could inhibit the transport of peptides through TAP [32]. Thus, we conclude that there may be some critical mechanisms used by DPV CHa strain to evade MHC class I molecules identify and presentation and then finally evade immune response.

Although MHC class I and MHC class II are similar in presenting virus antigen peptides to CD8^+^ and CD4^+^, the basic antigen presentation pathways of them are extremely diverse [28]. Firstly, these molecules have different cell distributions. MHC class I molecules are expressed at kinds of nucleated cells, however, MHC class II molecules are specifically found at antigen presenting cells (APCs), such as dendritic cells macrophages and B cells [33]. Unlike the source of viruses degraded in MHC class I molecules presentation pathway, virus protein presented in MHC II pathway is degraded in endosome. As for MHC class II molecules, after assembling with the transmembrane chains and the invariant chain (Ii, also named CD74) in the ER, MHC class II is transported to the MHC class II compartment (MIIC). In this part, Ii is digested by endosomal proteases such as asparaginyl endopeptidase (APE) and then leave a residual class II-associated Ii peptide (CLIP) occupying the peptide-binding groove of the MHC class II molecules [34]. In the MIIC, the CLIP fragment is displaced by viral protein segment through participation of the H2-DM chaperone [35]. The MHC II molecules then traffic to the plasma membrane and present virus peptides to CD4^+^ cells [35]. From our results, extreme diversity of different genes expression of MHC-II pathway has been present in CHv infection group and CHa vaccinated group (Table 3). The expression of genes related to basic composition of MHC-II like CD74 was up-regulated in CHv infected group which means the infection of CHv strain processes the potential to be presented to CD4^+^ cells and then evoke duck adaptive immune response in 24 hpi. Yet,genes like MHC2 and GILT related to CHa vaccinated group were down-regulated. GILT, a gamma-interferon inducible lysosomal thiol reductase, could catalyze antigenic disulfide bond reduction in endocytosis pathway and this function is essential in MHC-II antigen processing [36]. From our results, we conclude that in order to evade immune response, CHa depressed the expression of GILT and escape the degradation of disulfide bond. It may be an effective way for CHa to avoid identification by CD4^+^ cells. Meanwhile, we also found that, no matter in CHv infected group or CHa vaccinated group, the expression of Class II Transcriptional Activator (CIITA) gene was down-regulated. Previous works reported that, CIITA is a co-activator and tightly controls expression of all components of MHC-II at the transcriptional level, such as MHC-II and Ii [37]. It means that CIITA plays an important role in MHC-II antigen processing in both CHv and CHa infection.

From this part, we conclude that CHv could be present by MHC-I and MHC-I as well as identified by both CD8^+^ T cells and CD4^+^ T cells, and as for CHa, there may be some critical ways to escape the immunosurveillance at 24 h post DPV infection.

### Cytokine–cytokine receptor interaction

Cytokines are soluble extracellular proteins or glycoproteins including chemokines, interferons (IFN), interleukins (IL), lymphokines, and tumor necrosis factors (TNF) [38]. Cytokines play crucial roles in many cell biological processes. They are engaged in humoral and cell-based immune responses, cell growth, differentiation, maturation, death, angiogenesis and homeostasis [38]. Through binding through corresponding cytokines receptors, cytokines transmit signals from outside into inside of cells. In cells, through phosphorylation and dephosphorylation events, signal transduction pathways are activated [38]. In our results, from cytokine–cytokine receptor KEGG pathway, we found that the expression of many important cytokines and cytokine receptors genes were changed in CHv infected or CHa vaccinated group. For example, the expression of IFN-γ receptor IFNGR1, the common receptor CSF2RB of IL3, IL5 as well as tumor necrosis factors receptor SF18 were depressed after CHv infection. The expression of PDGFRB genes involved in tyrosine kinase receptors and SF21 related to TNF receptors were increased in CHv infected group. Meanwhile, CX3CL1 and CCL19 chemotactic factor as well as transforming growth factor TGFB3 were up-regulated in CHa vaccinated group. Cytokines receptors including IL-6 receptor CNTFR as well as tyrosine kinase receptors FLT1 and FLT4 were also increased after CHa vaccination. Yet, the expression of IL22 receptor, IL22RA2 was down regulation in CHa vaccination. Furthermore, the expression of IL15 and TNFSF13B were down regulation and epidermal growth factor (EGF) receptor EGFR was up regulation in both CHv and CHa infected group. Some significant genes related to viral immune evasion were picked out and performed detailed illustrations.

Viral invasion of a host cell triggers immune responses and the major protection against viral infection implemented by host is activation of the interferon (IFN)-mediated antiviral pathway. IFNs, one of a family of cytokines, contain two subtypes including type I IFNs (IFN-α and IFN-β) and type II IFN (IFN-γ). IFN-α/β is responsible for the recognition and clearance of virus as the first innate immune defense. IFN-γ is produced by activate T cells and nature kill (NK) cells. It is involved in many biological processes including immunosurveillance and cell-mediated immune response [39]. From our results, we found that the expression of IFN-γ receptor IFNGR1 was down-regulated after CHv infection. It has been reported that Kaposi’s sarcoma-associated herpesvirus (KSHV) has been shown to inhibit the function of IFN-γ through depressing the expression of IFNGR1 with the help of K3 and K5 protein [40]. Similarly, another herpesvirus, Epstein–Barr virus (EBV), also block the process of IFN response through down regulation of the IFN-γ receptor gene expression by BZLF1 [41]. It means that just like another herpesvirus, highly virulence DPV CHv strain suppress both cytokines-induced and cell-mediated immunity response through depressing the expression of IFN-γ receptor. It may be an efficient way for CHv to avoid host immune control.

Chemokines are pivotal regulatory factors on the aspects of the accumulation, activation and movement of leukocytes into inflamed cells [38]. CX3CL1 is a cell surface-expressed chemokine and plays a distinct role in several aspects of leukocyte activation and motion during inflammation and viral infection [42]. KSHV encodes CX3CL1 receptor homologs protein US28 to prevent the function of CX3CL1 and then serve the immune evasion purpose [42]. In CHa vaccinated group, CX3CL1 gene was increased in expression. We conclude that CXC3L1 may perform an important role no matter in host immune defense or in viral immune evasion during CHa infection. Further studies need to be done to uncover the exact functions of CXC3L1 in CHa infection.

Nature killing (NK) cells are Innate Lymphoid Cells (ILC) and are capable of killing virus-infected cells in the early stage of infections. IL15, type I cytokines, possesses a pivotal biological activity in stimulating NK cells and controlling various aspects of NK cell immunologic surveillance process [43]. For instance, previous studied reported that IL15 stimulated NK cells cytotoxicity and then severely depress human herpesvirus-6 (HHV-6) expression [44]. This study showed that IL-5 gene expression was reduced after CHv and CHa infection. It means that depression of the expression of IL15 may be an effective way for DPV to evade NK cell immunologic monitor at 24 hpi.

### Apoptosis

Cells infected with viruses would occur apoptosis to protect other cells from infection and reduce production of new virus from these infected cells [45]. Therefore, in order to survive long enough in cells, viruses have evolved apoptotic inhibitory mechanisms. Successful inhibitory mechanisms alter host cell signaling pathways to govern cell survival and the viral life cycle.

Viruses selectively inhibit apoptotic pathways through blocking the synthetic processes such as depressing the synthesis of pro-apoptotic protein like caspases and p53 or the expression of anti-apoptotic protein like Bcl-2 [46, 47]. Furthermore, PI3K-Akt, an important anti-apoptosis signaling pathway, is commonly exploited by viruses to regulate cell survival [48]. Our results showed that the genes expression in PI3K-Akt signaling pathway was changed in CHv infected group (Table 3). It means that PI3K-Akt signaling pathway may be a key mechanism for highly virulence DPV CHv strain to regulate anti-apoptosis.

Phosphatidylinositol 3-kinase (PI3K) phosphorylates the lipid substrate PtdIns(4,5)P_2_ to PtdIns(3,4,5)P_3_ on the plasma membrane [49]. The phosphorylated lipid acts as a second messenger to activate Akt protein on plasma membrane. Activated Akt phosphorylates a number of target proteins, including transcription factor like NF-κB and pro-apoptotic protein caspase-9 and then lead to suppression of apoptosis [50]. Previous studied reported that PIK3/Akt pathway is also utilized by herpesvirus to maintain cellular survival and viral replication. For example, herpes simplex virus I (HSV-1) induced the phosphorylation of Akt and glycogen synthase kinase 3 (GSK-3) to regulate apoptosis and viral gene expression [48]. From our results, the PIK3CB gene expression was down-regulated after the infection of CHv. PIK3CB, one of the four catalytic subunits of PI3K, plays an important role in cell growth [51]. It means that regulating the expression of PIK3CB may be useful for CHv to anti-apoptosis. Furthermore, some other genes were also found related to apoptosis. IKKA, one of subunits of IkB kinase complex (IKK), from our results, was down regulated in both CHv infected group and CHa vaccinated group. The IKKA is necessary for IkB phosphorylation and NF-kB activation [52]. Moreover, the expression of CASP6, apoptosis-related cysteine protease, was also depressed in CHv infected group and CHa vaccinated group [53]. It means that IKKA and CASP6 genes play a critical role in modulating cell apoptosis in highly virulence CHv infected group and attenuated CHa vaccinated group.

### Viral invasion

With no autonomous metabolic and motile capacity, viruses must rely on host cells to achieve genome replication, protein synthesis and structural assembling [54]. In order to finish above processes, viruses must suffer from four steps of cell entry processes that is adsorption to the cell surface, penetration, intracellular transport and egress from nucleus [55].

Adsorption to the cell surface is regarded as a prelude to viral infection and cell surface specific structures serve as viral binding locus. Heparan sulfate proteoglycans (HSPGs) are glycoproteins and participate many biological systems, and one of the important roles for HSPGs is adsorption receptors and facilitates viruses concentrated on the cell surface. HSPGs are the first receptor for many herpes viruses entry process, such as HSV, varicella zoster virus (VZV) and pseudorabies virus (PRV), to attach around host cell surface through their envelope glycoproteins like gB and/or gC [56, 57]. However, the types of adsorption receptors of DPV on duck cells surface and the specific interactive envelope proteins of DPV have not been reported in recent years. In our results, the different gene expression of HSPG2 gene was detected in CHv infected ducks indicating that HSPG perhaps plays an important role in the cell surface adsorption process of DPV (Table 3). Interestingly, although Yong reported that DPV glycoproteins gC played an important role in the initial adsorption on chicken embryo fibroblasts (CEF) cell surface, however, they detected that HSPGs was not interactive sites to DPV gC [58]. Therefore, it is necessary to perform further experiments to explore the functions of HSPGs on the process of DPV entry and the types of DPV glycoprotein interacted with HSPGs.

The next necessary step for enveloped viral entry is penetration reaction and this membrane fusion process is promoted by cues of specific envelope-membrane receptor bindings [54]. Specific cellular receptors participated in penetration process of herpes virus, such as HSV, fall into three classes, and one of important receptors are specific sites in heparan sulphate catalyzed by certain heparan sulfate glucosamine 3-*O*-sulfotransferase (HS3ST) [59]. Nevertheless, so far there are still no relevant reports paid attention on the types of envelope-membrane binding receptors when ducks infected by DPV. In our study, we detected that the expression of HS3ST genes HS3ST5 was changed in CHv infected ducks (Table 3). It suggests that for highly virulence strain of DPV, HS3ST protein may exercise as the same functions as other herpes virus.

In order to finish viral replication, assembly and maturation, intracellular complexes of virus utilize host intracellular cytoskeletal components and move from cell periphery to synthesis sites [54]. After assembly and maturation in cell, viral particles move from cell center to the cell membrane [54, 60]. Microtubules and actins, two important parts of cell cytoskeleton, play significant roles in herpesvirus transport within cells [61]. For example, microtubules provide long-distance transport for HSV with the assistance of their motor protein, kinesin. Our results showed that the expression of two types of alpha-tubulin (TUBA1C and TUBA1B) gene, whose products are basic components of microtubules, was changed in CHv infected ducks. Notably, the expression of kinesin-related genes were also found changed in CHa vaccinated ducks. N-type kinesin, one of a microtubules-binding plus-end-directed motors, could transport viral particles from their replication and transcription sites to cell membrane [62]. KIF1A, one of a member of N-types of kinesins, plays a key role in HSV transport, and its co-localization with herpes simplex virus II (HSV-2) UL56 protein were also reported [61]. In our study, a N-types kinesin gene KIF14 was detected  different expression in CHa infected ducks (Table 3). It suggests that CHa strain could rely on important motors of microtubules to finish its cellular long-distance movement just like other herpes virus. In addition to microtubules, another medium, actin filament, is essential for herpesvirus to finish short-range transport [61]. It also contains fast-growing plus-ends and slow-growing minus-ends to link cell center and cell surface [61]. Myosin is a crucial motor to mediate the movement of HSV along actin filaments [61]. Our results also found the expression of some actin-related genes and myosin-related genes to give expression changes in both CHv and CHa infected duck (Table 3), indicating that short-distance transport is an important way to complete intercellular movement process of DPV and some relevant proteins are also used to finish this process.

Although the mechanisms by which DPV interacts with cell surface receptor to achieve viral adsorption, membrane fusion and motors have not been clearly studied, several significant genes related to DPV invasion were detected in this results and it may provide an initial view and supply a foundational data support for future experimental researches in DPV invasion aspect.

## Abbreviations

DPV: duck plague virus
CHv: DPV Chinese virulent strain
CHa: attenuated DPV vaccines
HSV: herpes simplex virus
BHV-1: bovine herpes virus
HCMV: human cytomegalovirus
KSHV: Kaposi’s sarcoma-associated herpesvirus
EBV: Epstein–Barr virus
HHV-6: human herpesvirus-6
VZV: varicella zoster virus
PRV: pseudorabies virus
DEGs: differentially expressed genes
GO: gene ontology
BP: biological proces
CC: cellular component
MF: molecular function
CTLs: cytotoxic T lymphocytes
ER: endoplasmic reticulum
β_2_m: β_2_-microglobulin
TAP: transporter associated with Antigen Processing
APCs: antigen presenting cells
MIIC: MHC class II compartment
APE: asparaginyl endopeptidase
CLIP: class II-associated Ii peptide
CIITA: class II transcriptional activator
IFN: interferon
IL: interleukin
TNF: tumor necrosis factors
EGF: epidermal growth factor
ILC: innate lymphoid cell
PI3K: phosphatidylinositol 3-kinase
GSK-3: glycogen synthase kinase 3
HS3ST: heparan sulfate glucosamine 3-*O*-sulfotransferase
